# Genotypic characterization of rotavirus in children under 5 years circulating in Côte D’Ivoire from 2010 to 2013

**DOI:** 10.1186/s12985-018-0973-z

**Published:** 2018-04-27

**Authors:** Catherine Boni-cisse, Sindou Meite, Alice Britoh Mlan, Flore Zaba, Rebecca N’Guessan, Nicaise Aka Lepri, Bélinda Lartey

**Affiliations:** 1 UFR des Sciences Médicales, Département de Microbiologie, Université Félix Houphouët Boigny, Abidjan, Côte d’Ivoire; 2 Laboratory of Sentinel Site Surveillance of Paediatric Bacterial Meningitis and Rotavirus Diarrhoea CHU Yopougon, Abidjan, Côte d’Ivoire; 3 Paediatric Service CHU Yopougon, Abidjan, Côte d’Ivoire; 4 Expanded Program on Immunization of Côte d’Ivoire, Abidjan, Côte d’Ivoire; 5 NMIMR West African Regional Rotavirus Reference Laboratory, Accra, Ghana

**Keywords:** Rotavirus, Genotype, Surveillance, Côte d’Ivoire

## Abstract

**Background:**

Rotavirus infection is the most common cause of severe gastroenteritis in children under five years of age in both developed and developing countries. The World Health Organisation (WHO) recommends the surveillance of rotavirus strains prior to vaccine introduction in all applicable countries. The objective of this study was to describe the epidemiological characteristics as well as to determine the circulating genotypes of rotaviruses in Côte d’Ivoire prior to vaccine introduction.

**Methods:**

The study included children under five years of age who met the inclusion criteria after informed consent had been sort from their parents or guardians. Rotavirus VP6 antigens were detected for each stool sample using Enzyme Immunoassay (EIA). Genotyping of positive EIA samples was performed by reverse-transcriptase-PCR (RT-PCR) assays.

**Results:**

A total of 684 children were recruited. Children aged between 6 and 11 months were the most represented with 34%. Rotavirus VP6 antigens were found in 27.1% (186/684) of samples tested. Commonly detected G genotypes included G12 (46.6% (82/176) and G1 (13.1% (23/176) whilst P[8] (49.8% (91/183) was the most predominant P genotype. Rotavirus G12P[8] was the most predominant strain circulating in Côte d’Ivoire within the period of study and constituted 26.6% of all strains detected.

**Conclusion:**

The monitoring of circulating strains will help guide decision-makers in the choice of vaccine. Genotypic variability of circulating rotavirus strains over the years implies there is a need for continuous rotavirus strain surveillance even after vaccine introduction.

## Background

The rotavirus, a non-enveloped wheel-like virus with 11 double stranded (ds) RNA segments, is responsible for acute gastroenteritis characterized by the sudden onset of watery diarrhoea, fever and vomiting [1]. Group A rotaviruses are the single most common cause of severe gastroenteritis and consequent dehydration in young children both in the developing and industrialized countries [2]. They are responsible for approximately 200,000 deaths globally with a greater proportion of these deaths occurring in sub-Saharan Africa and South East Asia [3]. To help reduce the high disease burden associated with rotavirus diarrhoea, the World Health Organisation (WHO) in 2009 recommended the global use of rotavirus vaccines within the national immunization programmes of countries [4]. Presently, two oral, live attenuated rotavirus vaccines; Rotarix® a monovalent rotavirus vaccine and RotaTeq™ a pentavalent rotavirus vaccine have been licensed for universal use [5, 6]. Both vaccines have been shown to provide homotypic and heterotypic protection. The use of these vaccines in Europe, Latin America and Africa have shown remarkable decline in infantile morbidity and mortality due to rotavirus associated diarrhoea [7].

Rotavirus outer VP7 and VP4 capsid genes provide the basis for a dual classification system. Molecular typing of these genes have provided vast epidemiological information on the diversity of rotavirus strains in circulation globally [8]. Currently, 27 G-genotypes and 37 P-genotypes have been described worldwide [9]. However, five most common genotype combinations including G1P[8], G2P[4], G3P[8], G4P[8] and G9P[8] are known to account for approximately 75% of all severe rotavirus infections in humans [10]. Nevertheless, a recent WHO surveillance report noted the predominance of uncommon strains including G12P[8], G12P[6]; G2P[6], G3P[6], G1P[6]; G1P[4], G2P[8]; and G9P[4] circulating in South East Asia; sub-Saharan Africa; West Pacific and the Americas respectively [11].

With the effect of rotavirus vaccines on the natural pattern of circulating rotavirus strains within the human population unknown and difficult to predict, WHO recommends continuous surveillance for the documentation of temporal changes in rotavirus strains before and after vaccine introduction in countries.

Rotavirus surveillance in Côte d’Ivoire began with WHO support under the supervision of the Expanded Program on Immunization (EPI) in 2010 to monitor circulating rotavirus strains as well as study the transmission dynamics of the virus. The epidemiological data generated from this surveillance study would be essential to guide recommendations in the choice of rotavirus vaccine. This study describes the epidemiological characteristics and distribution of circulating rotavirus G- and P-genotypes isolated from Ivorian children under five years of age with acute gastroenteritis pre-vaccine introduction era.

## Methods

### Study population and sample collection

The study included children less than 5 years of age hospitalized or being treated for acute gastroenteritis (acute diarrhea (< 14 days) in any one of the rotavirus sentinel surveillance sites Altogether, there were six sentinel sites located within five municipals in the city of Abidjan. Between the period January, 2010 and December 2013, diarrheic stool samples (without mucus or blood) were collected from participants for whom informed consent had been obtained from parents or guardians. Samples were collected from these children within 48 h of hospitalization. Socio-demographic and clinical information were also taken. Samples collected were stored in coolers containing refrigeration accumulators allowing temperatures between 0 and 4 °C. Samples were later transferred to the Bacteriology-Virology unit of the central laboratory of the Yopougon University Teaching Hospital where they were stored at 4 °C for a maximum of 30 days for serological analysis. The samples were then stored at − 20 °C until ready to be transported to the WHO Rotavirus Regional Reference laboratory in Ghana or South Africa.

### Laboratory analysis

#### Detection of group a rotavirus antigens

Samples were screened for the presence of rotavirus structural protein VP6 by the use of Rotaclone® a rapid EIA test kit following the manufacturers’ instructions. Samples with optical density > 0.25 at 450 nm wavelength were considered positive.

### Molecular characterisation of rotavirus strains

#### Polyacrylamide gel electrophoresis (PAGE)

To ascertain viral RNA integrity as well as screen for non-group A rotaviruses, all EIA rotavirus positive samples were further analysed by electrophoresing on polyacrylamide gel following genome viral RNA extraction by Bender buffer treatment and ethanol precipitation. The dsRNA segments were seperated by PAGE at 100 V for 18 h and bands visualised by silver-staining technique [12].

#### G- and P-genotyping assays

To determine the VP7 (G-) and VP4 (P-) genotypes, viral RNAs was extracted from the clarified supernatant of 20% stool suspensions using the QIAamp® Viral RNA Mini kit (QIAGEN®, Hilden, Germany) following the manufacturer’s instructions. Reverse transcription (RT)-PCR was performed using both forward and reverse consensus primers Beg9/End9 and Con3/Con2 to amplify a 1069 bp and 835 bp fragments of the VP7 and VP4 genes respectively. Multiplex PCR was carried out for G- and P-typing with genotype specific primers as previously described [8, 13, 14].

PCR amplicons were electrophoresed on a 2% agarose gel in Trisborate- EDTA buffer along with a 100-bp DNA ladder.

### Data analysis

All statistical analysis was performed with the EPI-Info version 3.5.4 software (CDC Atlanta, USA). All categorical variables were summarized as proportions, and significance of their difference in distribution with the outcome was assessed using Pearson’s chi-square and Fisher test at 5% risk.

## Results

**Fig. 1:**
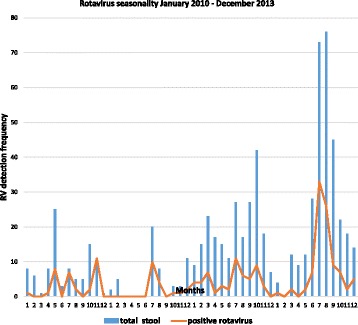
Monthly distribution of rotavirus-associated acute gastroenteritis from January 2010 to December 2013. Graph shows the peak of rotavirus detection in July each year and in November 2010

**Fig. 2:**
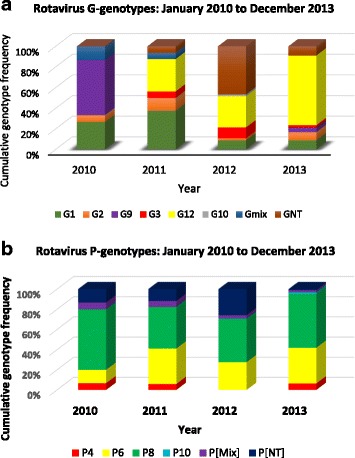
Temporal rotavirus genotype distribution in Côte d’Ivoire **a**. Rotavirus G-type distribution **b**. Rotavirus P-type distribution. Mix: multiple genotypes detected for either G, P or both; NT: either G, P or both were nontypeable

A total of 684 stool samples were collected from children hospitalized with acute gastroenteritis between January 2010 and December 2013 from six rotavirus sentinel sites in Abidjan, Côte d’Ivoire. The number of samples collected increased gradually over the four year study period, with sample numbers ranging from 91 samples in 2010 to 313 samples in 2013 (Table 1). The decrease in samples collected in 2011 was due to the civil war which took place in the country during that period. The study population consisted of a male to female ratio of 1:4.3. Of the children hospitalized with diarrhoea, 34% (233/684) were in the age group 6 to 11 months, while 26% (178/684) were ≤ 6 months. An overall rotavirus positivity of 27.1% (186/684) was observed over the four year study period. Rotavirus positivity rates however varied over the years and ranged from 16.4 to 34.6% (Table 1). Overall, rotaviruses were detected from stool samples collected in each month of the study period except for the months of March to June 2011 when sample collection was hindered by the civil war. The peak for rotavirus infection was in the month of July for each study year and in November, 2010 (Fig. 1). Using multiplex RT-PCR assay, we could assign both G- and P-genotypes to 113 of 186 (60.8%) EIA positive samples whilst 37 (19.9%) and 19 (10.2%) positive samples could be assigned a G- or P-genotype respectively. Sixteen (16/186; 8.6%) rotavirus strains remained untyped for both VP7 and VP4 genes (Table 2). Commonly circulating VP7 genotypes detected during the entire study period (2010–2013) included G12 (44.1%) the most predominant, followed by G1 (9.1%), G9 (6.5%), G2 (5.9%) and G3 (4.3%). Similarly, rotavirus P [8] and P[6] VP4 genotypes were the most predominant (44.1%; 34.0% respectively) P-types circulating during the study period. Genotypes G8 and P[4] as well as mixed infections were less frequently detected and constituted less than 2% of isolated genotypes. Throughout the study period, rotavirus genotype G12 was most prevalent expect in the year 2010 when G9 was the most predominant genotype (Fig. 2a). Genotypes G1, G2 and G3 were detected throughout the study period at varying frequency. On the other hand, there was no change in the predominant P-type as P [8] remained dominant over the study period (Fig. 2b). The uncommon P [10] P-type was detected in a single sample in 2013. G12 P[8] (27.4%), G12P[6] (15.6%), G1P[8] (5.4%) and G3P[6] (3.8%) were the most prevalent rotavirus strains associated with acute gastroenteritis in Ivorian children.

**Table 1:** Socio-demographic characteristics of study participants

Year					
Variable	2010	2011	2012	2013	Total
	N (%)	N (%)	N (%)	N (%)	N (%)
Sex					
Female	41(45)	26(50)	83(36.4)	131(41.8)	281(41.1)
Male	50(55)	26(50)	145 (63.6)	182(58.2)	403(58.9)
Total	91(100)	52(100)	228 (100)	313(100)	684(100)
Age (Months)					
0–5	20(22)	13 (25)	66(29)	82(26)	181(26)
6–11	28(31)	16(31)	74(32)	112(36)	230(34)
12–17	19(21)	11(21)	49(21)	57(18)	136(20)
18–23	12(13)	7(13)	18(8)	31(10)	68(10)
> 24	12(13)	5(10)	21(10)	31(10)	69(10)
Total	91(100)	52(100)	228(100)	313(100)	684(100)
EIA Result					
Positive	15(16.4)	18(34.6)	59(25.9)	94(30)	186(27.1)
Negative	76(83.6)	34(65.4)	169(74.1)	219(70)	498(72.9)
Total	91(100)	52(100)	228(100)	313(100)	684(100)

*N* Number or Frequency

**Table 2:** Rotavirus strain distribution between the period January 2010 and December 2013

VP7-Type	VP4-Type					
	P[4]	P[6]	P[8]	P[Mix]	P[NT]	Total (%)
G1	0	1	10	0	6	17 (9.1)
G2	2	5	0	1	3	11 (5.9)
G3	0	7	1	0	0	8 (4.3)
G8	0	0	1	0	0	1 (0.5)
G9	0	4	0	0	8	12 (6.5)
G12	0	29	51	1	1	82 (44.2)
GMix	0	0	0	0	1	1 (0.5)
GNT	0	17	19	1	16	36 (19.3)
Total (%)	2 (1.1)	63 (33.9)	82 (44.1)	3 (1.6)	36 (19.3)	186 (100)

*GMix / P[Mix]*: multiple genotypes detected for either G, P or both; *GNT / P[NT]*: either G, P or both were none typeable

## Discussion

Rotavirus gastroenteritis associated with severe dehydration mainly affect the population of children under five years and especially before the age of 1 year both in the developed and developing countries. The WHO therefore recommends the administration of several doses of rotavirus vaccines to children from 4 to 6 weeks old [15]. In countries like Côte d’Ivoire where rotavirus vaccine is yet to be introduced, monitoring of rotavirus disease as well as circulating strains would be necessary to assess the impact of the vaccine on rotaviruses when it is introduced. This study provides data for sentinel monitoring of rotavirus diarrhoea from 2010 to 2013 pre-vaccine introduction era in Côte d’Ivoire. Children under 12 months of age were the most susceptible to gastroenteritis and this observation compares with previous studies carried out in Côte d’Ivoire from 2000 to 2010 [16, 17]. In sub-Saharan Africa more than 40% of cases of acute diarrhoea are attributed to rotaviruses with a high prevalence in children under 2 years of age [18]. During the four year study period, the prevalence of rotavirus associated diarrhoea of 27.1% was significantly lower, but comparable to pre-monitoring studies conducted in Uganda and elsewhere in Côte d’Ivoire that reported rotavirus prevalence of 28.9% from 2000 to 2004 and 28. 6% from 2007 to 2009 respectively [16, 19]. In other west African countries however, prevalence ranging from 34 to 48% have been observed [20, 21] A study on the viral etiology of acute gastroenteritis in Burkinabe children under five found that rotaviruses were predominant with 63.5% prevalence rate [22]. Peak of viral infection was observed between July and August corresponding to the rainy and wet season as already not established. Although several studies from sub-Saharan African countries point for a higher prevalence of RVA infection in the dry season [23]. Rotavirus diarrhoea is mainly associated with five genotypes; G1, G2-G4, G9 and P[8] with G1P[8] genotype combination commonly associated with infections worldwide [24]. Over the four year period of study, temporal fluctuations and variability in the frequency of rotavirus genotype combinations was observed from one rotavirus season to the other. The study identified six G- (G1, G2, G3, G8, G10, G12) and 4 P-genotypes (P[8], P[6], P[4], P[10]) occurring in diverse genotype combinations. Interestingly, though rotaviruses with the common G1P[8] genotype combination were associated with infections over the entire study period at varying frequencies, the atypical rotavirus strains G12P[8] and G12P[6] were the most prevalent and together were responsible for over 43% of all rotavirus infections in Ivorian children. Formally sporadically detected, recent publications have described this G12 genotype as important emerging agents of acute gastroenteritis [25]. This genotype was also isolated in Gabon and some parts of Europe around the same period as this study [26]. It however remains unclear if these G12 genotypes are transient or will become established within our settings. Close association of humans with domesticated animals in most countries of the developing region lead to gene reassortment events within commonly circulating rotavirus strains thus giving rise to a large genomic diversity and frequent occurrence of mixed infections [27]. Unlike previous reports of high percentage of mixed genotype detection [28, 29], this study observed less than 2% of mixed genotypes circulating within the study population. Findings from this present study represents pre-vaccination data that may be useful in future when assessing the effectiveness of any introduced rotavirus vaccine. Knowledge on genotype distribution over the years will also enable a better monitoring of the epidemiological evolution of rotavirus strains.

## Conclusion

Rotavirus diarrhoea remains a public health problem in Côte d’Ivoire hence the reason for sentinel surveillance. This study highlight the genotypic variability of circulating rotavirus strains before the introduction of rotavirus vaccines in the expanded immunization program. Continuous surveillance will be necessary to monitor prevalent and newly evolving rotavirus stains within the Ivorian community post vaccine introduction.
